# 3-(5-Chloro­naphthalene-1-sulfonamido)-2-(2-hy­droxy­eth­yl)-4,5,6,7-tetra­hydro-2*H*-pyrazolo­[4,3-*c*]pyridin-5-ium chloride

**DOI:** 10.1107/S1600536811012967

**Published:** 2011-04-13

**Authors:** Xiao-Guang Bai, Hong-Tao Liu, Yu-Cheng Wang, Ju-Xian Wang

**Affiliations:** a Institute of Medicinal Biotechnology, Chinese Academy of Medical Sciences and Peking Union Medical College, Beijing 100050, People’s Republic of China

## Abstract

In the cation of the title compound, C_18_H_20_ClN_4_O_3_S^+^·Cl^−^, the tetra­hydro­pyridinium ring assumes a half-chair conformation. The dihedral angle between the pyrazole ring and the naphthalene ring system is 75.19 (6)°. In the crystal, ions are linked into a three-dimensional network by N—H⋯O, N—H⋯Cl and O—H⋯Cl hydrogen bonds and weak π–π stacking inter­actions with centroid–centroid distances of 3.608 (2) Å.

## Related literature

For general background to potential anti­cancer kinase inhibitors, see: Fancelli *et al.* (2005 ▶); Gadekar *et al.* (1968 ▶). For a related structure, see: Brehm (1982 ▶).
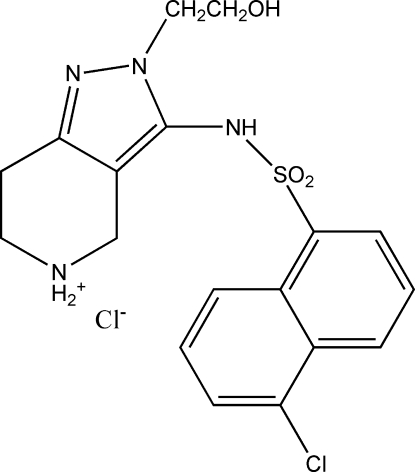


## Experimental

### 

#### Crystal data

C_18_H_20_ClN_4_O_3_S^+^·Cl^−^
*M* *_r_* = 443.34
Monoclinic, 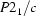

*a* = 14.790 (3) Å
*b* = 10.432 (2) Å
*c* = 13.155 (3) Å
β = 103.84 (3)°
*V* = 1970.9 (7) Å^3^
*Z* = 4
Mo *K*α radiation
μ = 0.46 mm^−1^
*T* = 294 K
0.25 × 0.20 × 0.18 mm

#### Data collection

Rigaku SCXmini diffractometer
19975 measured reflections
4511 independent reflections
3748 reflections with *I* > 2σ(*I*)
*R*_int_ = 0.036

#### Refinement

*R*[*F* ^2^ > 2σ(*F* ^2^)] = 0.041
*wR*(*F* ^2^) = 0.097
*S* = 1.08
4511 reflections
258 parameters
H atoms treated by a mixture of independent and constrained refinement
Δρ_max_ = 0.33 e Å^−3^
Δρ_min_ = −0.33 e Å^−3^

### 

Data collection: *CrystalClear* (Rigaku, 2005 ▶); cell refinement: *CrystalClear*; data reduction: *CrystalClear*; program(s) used to solve structure: *SHELXS97* (Sheldrick, 2008 ▶); program(s) used to refine structure: *SHELXL97* (Sheldrick, 2008 ▶); molecular graphics: *SHELXTL/PC* (Sheldrick, 2008 ▶); software used to prepare material for publication: *SHELXTL/PC*.

## Supplementary Material

Crystal structure: contains datablocks I, global. DOI: 10.1107/S1600536811012967/rz2578sup1.cif

Structure factors: contains datablocks I. DOI: 10.1107/S1600536811012967/rz2578Isup2.hkl

Additional supplementary materials:  crystallographic information; 3D view; checkCIF report

## Figures and Tables

**Table 1:** Hydrogen-bond geometry (Å, °)

*D*—H⋯*A*	*D*—H	H⋯*A*	*D*⋯*A*	*D*—H⋯*A*
N1—H1⋯Cl2	0.80 (2)	2.47 (2)	3.261 (2)	170 (2)
N3—H3*B*⋯O3^i^	0.90	1.97	2.814 (3)	155
N3—H3*A*⋯Cl2^ii^	0.90	2.26	3.1060 (19)	156
O3—H3*C*⋯Cl2^iii^	0.82	2.30	3.1151 (18)	172

Symmetry codes: (i) 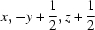; (ii) 
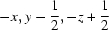
; (iii) 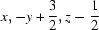.

**Fig. 1.**
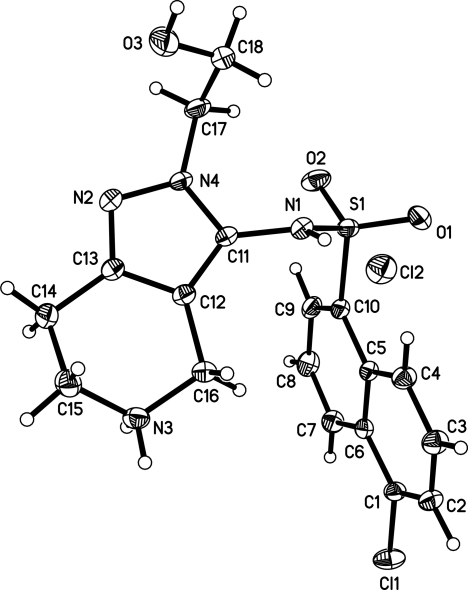
The molecular structure of the title compound, showing the atomic numbering scheme. Displacement ellipsoids are drawn at the 30% probability level.

**Fig. 2.**
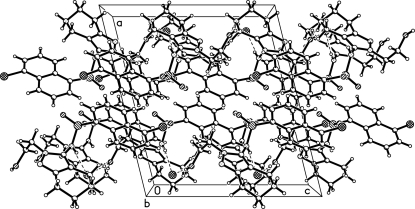
Packing diagram of the title compound viewed along the b axis. Hydrogen bonds are shown as dashed lines.

## supplementary crystallographic information

### Comment

As part of our ongoing project aimed at the development of potential anticancer kinase inhibitors (Fancelli *et al.*, 2005; Gadekar *et al.*, 1968), we have synthesized the title compound and report its crystal structure herein.

In the title compound (Fig. 1) bond lengths and angles have normal values. The dihedral angle between the planes of the pyrazole ring and the naphthalene ring system is 75.19 (6)°. As already observed in the related compound 2-methyl-4,5,6,7-tetrahydro-pyrazolo(3,4-c)pyridin-3-ol monohydrate (Brehm, 1982) the tetrahydropyridinium ring assumes a half-chair conformation, with atom C15 displaced by 0.665 (2) Å from the mean plane through the C12/C13/C14/C16/N3 atoms. The crystal structure (Fig. 2) is stabilized by N—H···O, N—H···Cl and O—H···Cl hydrogen bonds (Table 1) and by weak π···π stacking interactions occurring between centrosymmetrically related C5—C10 rings (centroid-to-centroid distance = 3.608 (2) Å).

### Experimental

A mixture of *tert*-butyl 3-cyano-4-oxopiperidine-1-carboxylate (22.4 g, 100 mmol) and 2-hydroxyethylhydrazine (7.76 g, 100 mmol) in ethanol was stirred at room temperature for 24 h and concentrated by evaporation, then crystallized in AcOEt and petroleum ether (5:1 *v*/*v*) to give *tert*-butyl 3-amino-2-(2-hydroxyethyl)-6,7-dihydro-2*H*-pyrazolo[4,3-*c*]pyridine-5(4*H*)-carboxylate as a yellow solid (13.5 g, one-step yield: 72%). A mixture of a portion (4.0 g, 14.2 mmol) of the material so obtained, azabenzene (10 ml) and dry acetonitrile was stirred at room temperature for 10 min and then a solution of 5-chloronaphthalene-1-sulfonyl chloride (3.7 g, 14.2 mmol) in dry acetonitrile (100 ml) was slowly added dropwise, stirred for 2 additional hours and concentrated by evaporation. The residue was purified by flash chromatography to give 3.9 g of *tert*-butyl 3-(1-chloronaphthalene-5-sulfonamido)-2-(2-hydroxyethyl)-6,7-dihydro-2*H*-pyrazolo[4,3-*c*]pyridine-5(4*H*)-carboxylate as a slightly yellow solid (one-step yield: 54%). Then, dry HCl gas was bubbled in a solution of the material so obtained (3.0 g, 5.9 mmol) in dry DCM (100 ml) for 3 h to get a white solid of the title compound (one-step yield:98%). Colourless block crystals suitable for X-ray diffraction were obtained in 5 days by slow evaporation of a methanol solution.

### Refinement

All H atoms were detected in a difference map. The H-atom bonded to N1 was refined freely, all other H-atoms were placed in calculated positions and refined using a riding motion approximation, with C—H=0.93–0.97 Å, with *U*_iso_(H)=1.2*U*_eq_(C); N—H=0.90 Å, with *U*_iso_(H)=1.2*U*_eq_(N); O—H=0.82 Å, with *U*_iso_(H)=1.5*U*_eq_(O).

### Crystal data

**Table:**

C_18_H_20_ClN_4_O_3_S^+^·Cl^−^	*F*(000) = 920
*M**_r_* = 443.34	*D*_x_ = 1.494 Mg m^−^^3^
Monoclinic, *P*2_1_/*c*	Mo *K*α radiation, λ = 0.71073 Å
Hall symbol: -P 2ybc	Cell parameters from 17906 reflections
*a* = 14.790 (3) Å	θ = 3.1–27.6°
*b* = 10.432 (2) Å	µ = 0.46 mm^−^^1^
*c* = 13.155 (3) Å	*T* = 294 K
β = 103.84 (3)°	Prism, colorless
*V* = 1970.9 (7) Å^3^	0.25 × 0.20 × 0.18 mm
*Z* = 4	

### Data collection

**Table:**

Rigaku SCXmini diffractometer	3748 reflections with *I* > 2σ(*I*)
Radiation source: fine-focus sealed tube	*R*_int_ = 0.036
graphite	θ_max_ = 27.5°, θ_min_ = 3.1°
Detector resolution: 13.6612 pixels mm^-1^	*h* = −19→19
CCD Profile fitting scans	*k* = −13→13
19975 measured reflections	*l* = −17→16
4511 independent reflections	

### Refinement

**Table:**

Refinement on *F*^2^	Primary atom site location: structure-invariant direct methods
Least-squares matrix: full	Secondary atom site location: difference Fourier map
*R*[*F*^2^ > 2σ(*F*^2^)] = 0.041	Hydrogen site location: inferred from neighbouring sites
*wR*(*F*^2^) = 0.097	H atoms treated by a mixture of independent and constrained refinement
*S* = 1.08	*w* = 1/[σ^2^(*F*_o_^2^) + (0.0347*P*)^2^ + 1.1868*P*] where *P* = (*F*_o_^2^ + 2*F*_c_^2^)/3
4511 reflections	(Δ/σ)_max_ < 0.001
258 parameters	Δρ_max_ = 0.33 e Å^−^^3^
0 restraints	Δρ_min_ = −0.33 e Å^−^^3^

### Special details

**Table:**

Geometry. All e.s.d.'s (except the e.s.d. in the dihedral angle between two l.s. planes) are estimated using the full covariance matrix. The cell e.s.d.'s are taken into account individually in the estimation of e.s.d.'s in distances, angles and torsion angles; correlations between e.s.d.'s in cell parameters are only used when they are defined by crystal symmetry. An approximate (isotropic) treatment of cell e.s.d.'s is used for estimating e.s.d.'s involving l.s. planes.
Refinement. Refinement of *F*^2^ against ALL reflections. The weighted *R*-factor *wR* and goodness of fit *S* are based on *F*^2^, conventional *R*-factors *R* are based on *F*, with *F* set to zero for negative *F*^2^. The threshold expression of *F*^2^ > σ(*F*^2^) is used only for calculating *R*-factors(gt) *etc*. and is not relevant to the choice of reflections for refinement. *R*-factors based on *F*^2^ are statistically about twice as large as those based on *F*, and *R*- factors based on ALL data will be even larger.

### Fractional atomic coordinates and isotropic or equivalent isotropic displacement parameters (Å^2^)

**Table:**

	*x*	*y*	*z*	*U*_iso_*/*U*_eq_	
C1	0.33453 (13)	0.45892 (19)	0.58292 (14)	0.0301 (4)	
C2	0.27302 (14)	0.5562 (2)	0.57754 (16)	0.0352 (4)	
H2	0.2497	0.5769	0.6352	0.042*	
C3	0.24496 (15)	0.6255 (2)	0.48403 (17)	0.0370 (5)	
H3	0.2015	0.6909	0.4795	0.044*	
C4	0.28016 (14)	0.59890 (19)	0.39948 (16)	0.0339 (4)	
H4	0.2603	0.6459	0.3381	0.041*	
C5	0.34684 (13)	0.50006 (18)	0.40460 (14)	0.0266 (4)	
C6	0.37435 (13)	0.42646 (18)	0.49814 (14)	0.0266 (4)	
C7	0.44156 (14)	0.32761 (19)	0.50555 (16)	0.0349 (4)	
H7	0.4588	0.2794	0.5666	0.042*	
C8	0.48095 (15)	0.3027 (2)	0.42407 (17)	0.0388 (5)	
H8	0.5247	0.2374	0.4297	0.047*	
C9	0.45611 (14)	0.3748 (2)	0.33186 (16)	0.0341 (4)	
H9	0.4843	0.3582	0.2772	0.041*	
C10	0.39047 (13)	0.46961 (18)	0.32171 (14)	0.0280 (4)	
C11	0.22097 (13)	0.42683 (18)	0.09794 (13)	0.0268 (4)	
C12	0.16017 (13)	0.34578 (18)	0.13062 (14)	0.0285 (4)	
C13	0.13724 (13)	0.25285 (19)	0.05261 (14)	0.0302 (4)	
C14	0.07198 (15)	0.1447 (2)	0.05610 (16)	0.0380 (5)	
H14A	0.1068	0.0672	0.0801	0.046*	
H14B	0.0329	0.1290	−0.0133	0.046*	
C15	0.01232 (15)	0.1807 (2)	0.13058 (17)	0.0423 (5)	
H15A	−0.0311	0.2476	0.0994	0.051*	
H15B	−0.0235	0.1067	0.1426	0.051*	
C16	0.12047 (15)	0.3506 (2)	0.22543 (16)	0.0358 (5)	
H16A	0.1700	0.3633	0.2879	0.043*	
H16B	0.0771	0.4215	0.2195	0.043*	
C17	0.28554 (16)	0.4324 (2)	−0.06542 (15)	0.0401 (5)	
H17A	0.2941	0.3657	−0.1136	0.048*	
H17B	0.3466	0.4569	−0.0242	0.048*	
C18	0.24133 (19)	0.5456 (2)	−0.12688 (17)	0.0486 (6)	
H18A	0.2272	0.6100	−0.0797	0.058*	
H18B	0.2842	0.5828	−0.1640	0.058*	
Cl1	0.36605 (4)	0.37099 (6)	0.69801 (4)	0.04925 (16)	
Cl2	0.09941 (4)	0.74385 (6)	0.16877 (4)	0.04289 (15)	
N1	0.26065 (12)	0.54306 (17)	0.14066 (12)	0.0303 (4)	
N2	0.18020 (12)	0.27265 (16)	−0.02419 (12)	0.0344 (4)	
N3	0.07142 (12)	0.22688 (17)	0.23298 (13)	0.0351 (4)	
H3A	0.0351	0.2367	0.2785	0.042*	
H3B	0.1141	0.1665	0.2590	0.042*	
N4	0.23153 (11)	0.38037 (16)	0.00456 (12)	0.0303 (4)	
O1	0.37721 (11)	0.69457 (15)	0.23095 (12)	0.0442 (4)	
O2	0.42594 (10)	0.50706 (17)	0.14152 (11)	0.0452 (4)	
O3	0.15812 (13)	0.50853 (16)	−0.19966 (14)	0.0547 (4)	
H3C	0.1378	0.5694	−0.2378	0.082*	
S1	0.36955 (3)	0.56130 (5)	0.20470 (4)	0.03171 (13)	
H1	0.2261 (16)	0.595 (2)	0.1539 (18)	0.038 (7)*	

### Atomic displacement parameters (Å^2^)

**Table:**

	*U*^11^	*U*^22^	*U*^33^	*U*^12^	*U*^13^	*U*^23^
C1	0.0327 (10)	0.0344 (10)	0.0233 (9)	−0.0036 (8)	0.0072 (8)	0.0018 (8)
C2	0.0380 (11)	0.0405 (11)	0.0312 (10)	−0.0007 (9)	0.0165 (9)	−0.0034 (9)
C3	0.0389 (11)	0.0363 (11)	0.0384 (11)	0.0094 (9)	0.0145 (9)	0.0025 (9)
C4	0.0388 (11)	0.0335 (11)	0.0294 (10)	0.0055 (8)	0.0084 (8)	0.0035 (8)
C5	0.0286 (9)	0.0281 (9)	0.0228 (9)	−0.0045 (7)	0.0056 (7)	−0.0031 (7)
C6	0.0278 (9)	0.0264 (9)	0.0252 (9)	−0.0035 (7)	0.0053 (7)	−0.0017 (7)
C7	0.0376 (11)	0.0321 (11)	0.0334 (10)	0.0034 (9)	0.0052 (9)	0.0029 (8)
C8	0.0364 (11)	0.0356 (11)	0.0436 (12)	0.0088 (9)	0.0080 (9)	−0.0045 (9)
C9	0.0347 (10)	0.0374 (11)	0.0322 (10)	−0.0031 (9)	0.0120 (9)	−0.0107 (8)
C10	0.0299 (9)	0.0302 (10)	0.0236 (9)	−0.0053 (8)	0.0061 (7)	−0.0039 (7)
C11	0.0293 (9)	0.0314 (10)	0.0193 (8)	0.0013 (8)	0.0051 (7)	0.0001 (7)
C12	0.0293 (9)	0.0334 (10)	0.0229 (9)	0.0005 (8)	0.0061 (7)	0.0003 (7)
C13	0.0317 (10)	0.0331 (10)	0.0250 (9)	0.0005 (8)	0.0051 (8)	0.0003 (8)
C14	0.0434 (12)	0.0371 (11)	0.0314 (10)	−0.0074 (9)	0.0051 (9)	−0.0020 (9)
C15	0.0338 (11)	0.0494 (13)	0.0417 (12)	−0.0099 (10)	0.0052 (9)	0.0007 (10)
C16	0.0417 (11)	0.0387 (11)	0.0307 (10)	−0.0067 (9)	0.0161 (9)	−0.0011 (8)
C17	0.0450 (12)	0.0545 (14)	0.0240 (10)	−0.0091 (10)	0.0150 (9)	−0.0028 (9)
C18	0.0769 (17)	0.0394 (13)	0.0318 (11)	−0.0168 (12)	0.0175 (11)	−0.0037 (9)
Cl1	0.0558 (3)	0.0630 (4)	0.0308 (3)	0.0095 (3)	0.0141 (2)	0.0166 (2)
Cl2	0.0352 (3)	0.0493 (3)	0.0467 (3)	0.0017 (2)	0.0147 (2)	−0.0030 (2)
N1	0.0328 (9)	0.0324 (9)	0.0260 (8)	0.0004 (7)	0.0080 (7)	−0.0021 (7)
N2	0.0413 (9)	0.0368 (10)	0.0252 (8)	−0.0031 (8)	0.0081 (7)	−0.0040 (7)
N3	0.0327 (9)	0.0429 (10)	0.0324 (9)	−0.0021 (7)	0.0129 (7)	0.0029 (7)
N4	0.0354 (9)	0.0366 (9)	0.0208 (7)	−0.0030 (7)	0.0105 (7)	−0.0011 (6)
O1	0.0556 (10)	0.0372 (8)	0.0395 (8)	−0.0172 (7)	0.0107 (7)	0.0016 (7)
O2	0.0364 (8)	0.0710 (11)	0.0321 (8)	−0.0051 (7)	0.0160 (6)	−0.0020 (7)
O3	0.0698 (11)	0.0408 (9)	0.0483 (10)	−0.0040 (8)	0.0041 (9)	0.0094 (8)
S1	0.0341 (3)	0.0385 (3)	0.0240 (2)	−0.0096 (2)	0.00986 (19)	−0.00055 (19)

### Geometric parameters (Å, °)

**Table:**

C1—C2	1.354 (3)	C13—C14	1.492 (3)
C1—C6	1.421 (3)	C14—C15	1.514 (3)
C1—Cl1	1.7359 (19)	C14—H14A	0.9700
C2—C3	1.401 (3)	C14—H14B	0.9700
C2—H2	0.9300	C15—N3	1.499 (3)
C3—C4	1.365 (3)	C15—H15A	0.9700
C3—H3	0.9300	C15—H15B	0.9700
C4—C5	1.417 (3)	C16—N3	1.495 (3)
C4—H4	0.9300	C16—H16A	0.9700
C5—C6	1.425 (3)	C16—H16B	0.9700
C5—C10	1.429 (3)	C17—N4	1.460 (2)
C6—C7	1.419 (3)	C17—C18	1.491 (3)
C7—C8	1.362 (3)	C17—H17A	0.9700
C7—H7	0.9300	C17—H17B	0.9700
C8—C9	1.399 (3)	C18—O3	1.420 (3)
C8—H8	0.9300	C18—H18A	0.9700
C9—C10	1.370 (3)	C18—H18B	0.9700
C9—H9	0.9300	N1—S1	1.6404 (18)
C10—S1	1.7755 (19)	N1—H1	0.80 (2)
C11—N4	1.364 (2)	N2—N4	1.358 (2)
C11—C12	1.376 (3)	N3—H3A	0.9000
C11—N1	1.405 (2)	N3—H3B	0.9000
C12—C13	1.393 (3)	O1—S1	1.4305 (16)
C12—C16	1.501 (3)	O2—S1	1.4276 (16)
C13—N2	1.332 (2)	O3—H3C	0.8200
			
C2—C1—C6	122.48 (18)	N3—C15—C14	110.87 (17)
C2—C1—Cl1	118.61 (15)	N3—C15—H15A	109.5
C6—C1—Cl1	118.90 (15)	C14—C15—H15A	109.5
C1—C2—C3	119.12 (18)	N3—C15—H15B	109.5
C1—C2—H2	120.4	C14—C15—H15B	109.5
C3—C2—H2	120.4	H15A—C15—H15B	108.1
C4—C3—C2	121.36 (19)	N3—C16—C12	108.59 (16)
C4—C3—H3	119.3	N3—C16—H16A	110.0
C2—C3—H3	119.3	C12—C16—H16A	110.0
C3—C4—C5	120.39 (18)	N3—C16—H16B	110.0
C3—C4—H4	119.8	C12—C16—H16B	110.0
C5—C4—H4	119.8	H16A—C16—H16B	108.4
C4—C5—C6	119.01 (17)	N4—C17—C18	113.64 (19)
C4—C5—C10	124.15 (17)	N4—C17—H17A	108.8
C6—C5—C10	116.83 (17)	C18—C17—H17A	108.8
C7—C6—C1	122.28 (17)	N4—C17—H17B	108.8
C7—C6—C5	120.10 (17)	C18—C17—H17B	108.8
C1—C6—C5	117.58 (17)	H17A—C17—H17B	107.7
C8—C7—C6	120.58 (19)	O3—C18—C17	110.29 (18)
C8—C7—H7	119.7	O3—C18—H18A	109.6
C6—C7—H7	119.7	C17—C18—H18A	109.6
C7—C8—C9	120.46 (19)	O3—C18—H18B	109.6
C7—C8—H8	119.8	C17—C18—H18B	109.6
C9—C8—H8	119.8	H18A—C18—H18B	108.1
C10—C9—C8	120.36 (19)	C11—N1—S1	124.79 (14)
C10—C9—H9	119.8	C11—N1—H1	116.8 (17)
C8—C9—H9	119.8	S1—N1—H1	114.3 (17)
C9—C10—C5	121.65 (18)	C13—N2—N4	104.68 (15)
C9—C10—S1	116.49 (15)	C16—N3—C15	113.93 (16)
C5—C10—S1	121.66 (15)	C16—N3—H3A	108.8
N4—C11—C12	106.69 (16)	C15—N3—H3A	108.8
N4—C11—N1	122.70 (16)	C16—N3—H3B	108.8
C12—C11—N1	130.33 (17)	C15—N3—H3B	108.8
C11—C12—C13	105.04 (16)	H3A—N3—H3B	107.7
C11—C12—C16	130.83 (17)	N2—N4—C11	111.59 (15)
C13—C12—C16	124.09 (17)	N2—N4—C17	119.05 (15)
N2—C13—C12	111.99 (17)	C11—N4—C17	129.32 (17)
N2—C13—C14	124.74 (18)	C18—O3—H3C	109.5
C12—C13—C14	123.26 (17)	O2—S1—O1	120.15 (10)
C13—C14—C15	108.30 (17)	O2—S1—N1	107.08 (9)
C13—C14—H14A	110.0	O1—S1—N1	104.36 (10)
C15—C14—H14A	110.0	O2—S1—C10	106.63 (10)
C13—C14—H14B	110.0	O1—S1—C10	109.08 (9)
C15—C14—H14B	110.0	N1—S1—C10	109.19 (9)
H14A—C14—H14B	108.4		
			
C6—C1—C2—C3	2.1 (3)	C16—C12—C13—C14	−1.7 (3)
Cl1—C1—C2—C3	−178.14 (16)	N2—C13—C14—C15	−159.55 (19)
C1—C2—C3—C4	−1.7 (3)	C12—C13—C14—C15	20.4 (3)
C2—C3—C4—C5	−0.3 (3)	C13—C14—C15—N3	−50.1 (2)
C3—C4—C5—C6	1.9 (3)	C11—C12—C16—N3	−170.03 (19)
C3—C4—C5—C10	−177.03 (19)	C13—C12—C16—N3	12.4 (3)
C2—C1—C6—C7	177.47 (19)	N4—C17—C18—O3	−67.3 (2)
Cl1—C1—C6—C7	−2.3 (3)	N4—C11—N1—S1	−80.5 (2)
C2—C1—C6—C5	−0.4 (3)	C12—C11—N1—S1	106.4 (2)
Cl1—C1—C6—C5	179.77 (14)	C12—C13—N2—N4	−0.2 (2)
C4—C5—C6—C7	−179.49 (18)	C14—C13—N2—N4	179.75 (18)
C10—C5—C6—C7	−0.5 (3)	C12—C16—N3—C15	−43.7 (2)
C4—C5—C6—C1	−1.5 (3)	C14—C15—N3—C16	66.5 (2)
C10—C5—C6—C1	177.46 (16)	C13—N2—N4—C11	0.2 (2)
C1—C6—C7—C8	−177.31 (19)	C13—N2—N4—C17	−177.76 (17)
C5—C6—C7—C8	0.5 (3)	C12—C11—N4—N2	−0.1 (2)
C6—C7—C8—C9	0.3 (3)	N1—C11—N4—N2	−174.68 (17)
C7—C8—C9—C10	−1.3 (3)	C12—C11—N4—C17	177.55 (19)
C8—C9—C10—C5	1.3 (3)	N1—C11—N4—C17	3.0 (3)
C8—C9—C10—S1	176.29 (16)	C18—C17—N4—N2	102.0 (2)
C4—C5—C10—C9	178.50 (19)	C18—C17—N4—C11	−75.5 (3)
C6—C5—C10—C9	−0.4 (3)	C11—N1—S1—O2	50.79 (18)
C4—C5—C10—S1	3.8 (3)	C11—N1—S1—O1	179.18 (15)
C6—C5—C10—S1	−175.13 (13)	C11—N1—S1—C10	−64.30 (17)
N4—C11—C12—C13	0.0 (2)	C9—C10—S1—O2	3.30 (18)
N1—C11—C12—C13	174.01 (19)	C5—C10—S1—O2	178.24 (15)
N4—C11—C12—C16	−177.9 (2)	C9—C10—S1—O1	−127.85 (16)
N1—C11—C12—C16	−3.9 (4)	C5—C10—S1—O1	47.09 (18)
C11—C12—C13—N2	0.1 (2)	C9—C10—S1—N1	118.67 (16)
C16—C12—C13—N2	178.22 (18)	C5—C10—S1—N1	−66.39 (17)
C11—C12—C13—C14	−179.82 (18)		

### Hydrogen-bond geometry (Å, °)

**Table:**

*D*—H···*A*	*D*—H	H···*A*	*D*···*A*	*D*—H···*A*
N1—H1···Cl2	0.80 (2)	2.47 (2)	3.261 (2)	170 (2)
N3—H3B···O3^i^	0.90	1.97	2.814 (3)	155
N3—H3A···Cl2^ii^	0.90	2.26	3.1060 (19)	156
O3—H3C···Cl2^iii^	0.82	2.30	3.1151 (18)	172

Symmetry codes: (i) *x*, −*y*+1/2, *z*+1/2; (ii) −*x*, *y*−1/2, −*z*+1/2; (iii) *x*, −*y*+3/2, *z*−1/2.
